# The Relationship Between Erectile Dysfunction and Dyadic Adjustment, Couple Relationship Quality, and Intimacy: A Cross-Sectional Study

**DOI:** 10.3390/medicina61091590

**Published:** 2025-09-03

**Authors:** Dragoș-Mihail Trifu, Daniel-Corneliu Leucuța, Martina-Luciana Pintea-Trifu, Florin Elec, Nicolae Crișan, Dan Eniu, Ioan Coman

**Affiliations:** 1 Department of Urology, Iuliu Hațieganu University of Medicine and Pharmacy, 400012 Cluj-Napoca, Romania; trifu.dragos.mihail@elearn.umfcluj.ro (D.-M.T.); florinelec@elearn.umfcluj.ro (F.E.); drnicolaecrisan@elearn.umfcluj.ro (N.C.); coman_ioan55@yahoo.com (I.C.); 2 Department of Urology, Municipal Blaj Hospital, 515400 Blaj, Romania; 3 Department of Urology, Endoplus Clinic, 400165 Cluj-Napoca, Romania; 4 Department of Medical Informatics and Biostatistics, Iuliu Hațieganu University of Medicine and Pharmacy, 400349 Cluj-Napoca, Romania; 5 Department of Cellular and Molecular Biology, Iuliu Hațieganu University of Medicine and Pharmacy, 400349 Cluj-Napoca, Romania; pintea.trifu.martina@elearn.umfcluj.ro; 6 Clinical Institute of Urology and Renal Transplantation, Iuliu Hațieganu University of Medicine and Pharmacy, 400000 Cluj-Napoca, Romania; 7 Department of Urology, Municipal Cluj Hospital, Iuliu Hațieganu University of Medicine and Pharmacy, 400139 Cluj-Napoca, Romania; 8 Department of Surgical Oncology, Iuliu Hațieganu University of Medicine and Pharmacy, 400000 Cluj-Napoca, Romania; daneniu@elearn.umfcluj.ro

**Keywords:** relationship dynamics, erectile dysfunction, lower urinary tract symptoms, Dyadic Adjustment Scale

## Abstract

*Background and Objectives*: This study aimed to evaluate the association between relationship dynamics as measured by dyadic adjustment and factors such as erectile function and lower urinary tract symptoms, adjusting for relevant clinical characteristics. *Materials and Methods*: This cross-sectional study collected data from 94 males in relationships of at least 6 months and with a prostate volume equal to or higher than 30 cc. Lower urinary tract symptoms, erectile function, and relationship dynamics were assessed with the International Prostate Symptom Score (I-PSS), International Index of Erectile Function (IIEF), Dyadic Adjustment Scale (DAS). *Results*: We found significant positive correlations between DAS affective expressions (AEs) and erectile dysfunction duration; between IIEF general satisfaction and DAS dyadic adjustment (DA), dyadic consensus (DC), and dyadic cohesion (DH); and between prostate width and DAS DA and DC (all ρ ≈ 0.2–0.3, *p* < 0.05). Further multiple regression analyses adjusting for age, prostate width, and comorbidities showed that the associations between IIEF general satisfaction and DAS DA (*p* = 0.013) and DH (*p* = 0.008) remained significant, while the relationship with DAS DC (*p* = 0.051) was borderline. *Conclusions*: Our findings highlight that general sexual satisfaction, as measured with the IIEF, had a small but independent association with higher affective expressions, dyadic cohesion, and dyadic consensus in couples, which are key domains of dyadic adjustment, regardless of relationship duration, prostate width, and comorbidities. These results emphasize the importance of considering sexual satisfaction in the context of relationship quality and, therefore, involving the female partner in the assessment and treatment of erectile dysfunction.

## 1. Introduction

Lower urinary tract symptoms (LUTSs) and erectile dysfunction (ED) are prevalent urological conditions that significantly affect the male population’s quality of life. According to the International Continence Society (ICS), lower urinary tract symptoms (LUTS) encompass a range of symptoms affecting the urinary bladder, prostate, and urethra, typically categorized into voiding, storage, or post-micturition symptoms [1]. These symptoms are widespread—affecting an estimated 2.1 billion individuals globally [2]—and are a major reason for urological consultations due to their chronic and bothersome nature [2,3]. As many as 72.3% of adult men have reported experiencing at least one lower urinary tract symptom (LUTS) during their lifetime. Parallelly, ED affected over 150 million men globally in the 1990s [4], with prevalence estimates in 2025 of 322 million men worldwide [4]. The European male population prevalence ranges from 31.3% and 76.5% [5]. A growing body of evidence supports a direct association between LUTSs and ED [6,7]. Recent studies, including a national Polish study, have identified a strong correlation between LUTSs and ED. Men presenting with lower urinary tract symptoms (LUTSs) had a higher likelihood of experiencing erectile dysfunction (ED) compared to those without LUTSs [8,9]. This association remained significant even after adjusting for factors such as age, comorbidities, and lifestyle. Furthermore, the severity of LUTSs was associated with a lower quality of both sexual and overall life quality [10]. In long-term follow-up data from the Massachusetts Male Aging Study (MMAS), the incidence of erectile dysfunction (ED) was reported at 26 new cases per 1000 men annually, while a Dutch cohort study with a mean follow-up of 4.2 years reported an incidence rate of 19.2 per 1000 person-years [11,12]. However, research addressing this issue in the Romanian population is still scarce.

Men’s health, traditionally centered around urological issues such as ED and benign prostatic hyperplasia (BPH), is increasingly viewed through a multidisciplinary lens, incorporating physical and psychological well-being. These findings are supported by results showing a significant prevalence of mental health issues, such as depression and sleep disorders, in men with urological symptoms [13,14]. ED, for example, has been linked to metabolic syndrome (MetS) and identified as a predictor of cardiovascular events, while LUTS has been associated with increased risks of anxiety and depression [2,13,15]. Studies using the International Prostate Symptom Score (IPSS) and the Patient Health Questionnaire (PHQ-9) as tools showed that men with LUTS are significantly more prone to experience depression and insomnia [5,16]. This evidence underlines the importance of a multimodal approach in the evaluation and management of men presenting to the urologist, advocating the need for integrated screening strategies and specialist referrals where appropriate. Also, there is less evidence of relationship dynamics and sexual satisfaction regarding the male perspective suffering from ED. Relationship dynamics refer to the patterns of interaction, emotional exchange, and mutual adjustment between partners.

Dyadic Scale Adjustment (DAS) is a well-validated psychometric instrument developed by Spanier in 1976 to assess the quality of couples’ relationships, particularly in the context of marital satisfaction. It evaluates relationship dynamics that may influence sexual function, mental health, and treatment adherence [17]. DAS is applied in studies regarding relationship quality and health outcomes such as erectile function, mental health disorders, and treatment adherence in chronic illnesses [18].

This study aimed to evaluate the association between dyadic adjustment and factors such as erectile function and lower urinary tract symptoms, adjusting for relevant clinical characteristics.

## 2. Materials and Methods

### 2.1. Study Design and Setting

This cross-sectional study collected data from males by having them complete a questionnaire that included the International Index of Erectile Function (IIEF-15), IPSS, and DAS, following a urologic consultation that included anamnesis, physical examination, abdominal ultrasound, and occasional blood work (PSA = Prostate-Specific Antigen, testosterone, SHBG = Sex Hormone-Binding Globulin, LH = Luteinizing Hormone, FSH = Follicle-Stimulating Hormone, urine culture). The study was conducted between March 2024 and January 2025, including a total of 94 patients.

### 2.2. Participants

The participants were selected from Prof. Dr. Ioan Puscas City Hospital in Simleu Silvaniei, Romania, and a private practice at Regina Maria Clinic in Cluj-Napoca. Patients were consecutive adult men attending those urology outpatient clinics for non-urgent evaluation. The main reasons for presentation were LUTS/BPH assessment and/or sexual dysfunction, with a minority attending for routine prostate health checks (e.g., PSA screening, BPH follow-up). No healthy volunteers were enrolled.

#### 2.2.1. Inclusion Criteria

All selected subjects were adult urology outpatients coded as male in the medical record who self-reported being men; they had to be in a relationship for at least six months and had to consent to an in-depth evaluation. Sexual orientation was not an inclusion criterion. In this cohort, 93 participants reported a female partner and one reported a male partner. Furthermore, each patient had to have a prostate volume of at least 30 cubic centimeters (cc). Men with marital problems and sexual dysfunction were also included in the study. Men with hormonal issues were referred to an endocrinologist, but were included in the study nevertheless.

#### 2.2.2. Exclusion Criteria

Patients who did not consent were excluded from the study, as well as the ones who did not complete the data for all variables in the questionnaire. Patients consulting primarily for sexually transmitted infections, acute stone colic, or oncologic pathology were not recruited.

Males suspected of prostate cancer (with a PSA > 4 ng/mL) and UTIs (Urinary Tract Infections) were also excluded from the study because of the multiple confounding factors that could interfere with the pathophysiological mechanisms of sexual function.

### 2.3. Variables and Measurement

The evaluation of LUTS was performed by completing the IPSS, and that of relationship dynamics was assessed by completing the DAS. Issues regarding sexual dysfunction were addressed by completing the IIEF-15. Data concerning smoking, alcohol, hypertension, chronic ischemic cardiopathy, pelvic trauma, obesity, and diabetes were collected through yes or no questions. The participants were also asked about their daily medication and had to name it.

An ultrasound was performed to assess the volume of the prostate with a 3.5 MHz transducer. The volume of the prostate was estimated by acquiring three anatomical dimensions. Width (W), measured laterally, and height (A), measured anteroposteriorly, were obtained in the transverse plane, while length (L) was measured from apex to base in the sagittal plane. We used the ellipsoid formula 0.52 × W × A × L to obtain the volume [19].

IPSS is a survey consisting of eight questions, seven of which are about symptoms and one of which focuses on quality of life (QoL). Symptom severity was classified based on the following scoring criteria: asymptomatic (0 points), mild (1–7 points), moderate (8–19 points), and severe (20–35 points) [20].

IIEF-15 is a well-validated psychometric instrument designed to appraise various domains of male sexual function. It consists of 15 items that evaluate erectile function (Questions 1–5 and 15), intercourse satisfaction (Questions 6–8), orgasmic function (Questions 9–10), sexual desire (Questions 11–12), and overall sexual satisfaction (Questions 13–14) [21]. The erectile function scoring is as follows: For the final statistical analysis, all 15 items were included, each rated from 1 to 5 points, rather than limiting the analysis to erectile function. It is essential to mention that higher scores indicate better sexual health.

DAS consists of 32 items organized into four subscales: dyadic consensus (DC) agreement between partners on subjects like recreation, finances, and affection; dyadic satisfaction (DS)—emotional satisfaction and conflict resolution within the relationship; dyadic cohesion (DH)—the degree of emotional bonding and shared activities; affectional expression (AE)—physical affection and sexual satisfaction. Every item is scored on a Likert-type scale with a total score ranging from 0 to 150—dyadic adjustment (DA). Higher scores mean better dyadic adjustment for couples [22]. The Dyadic Adjustment Scale (DAS) has been previously validated in Romanian populations. In particular, Turliuc and Muraru reported a three-factor structure (consensus, satisfaction, cohesion) accounting for 65.7% of the total variance and demonstrated adequate model fit and measurement invariance across genders in a sample of 383 Romanian married adults. Its internal consistency as measured by Cronbach’s α was as follows: DAS DA 0.90, DAS DC 0.81, DAS DS 0.85 and DAS DH 0.80. The Guttman split-half reliability coefficient for the DAS DA was 0.94. Furthermore, in a clinical sample, the DAS was described as having been validated and standardized for use in the Romanian population [23] (lines 156–162).

At enrolment we did not administer separate items distinguishing sex assigned at birth from current gender identity. The instruments (IIEF-15, DAS) use partner-neutral wording and were administered uniformly.

Also, all the questionnaires were completed by each participant.

### 2.4. Statistical Analyses

Descriptive statistics were calculated to summarize the characteristics of the study population. Categorical variables are presented as counts and percentages, while continuous variables are reported as medians with interquartile ranges (IQRs) due to non-normal distributions assessed visually with quantile–quantile plots. Associations between continuous variables were explored using the Spearman correlation coefficient (ρ), given the non-normality of the data. To explore the latent structure underlying the IIEF and DAS subscales, we conducted an exploratory factor analysis (EFA). The sampling adequacy was evaluated using the Kaiser–Meyer–Olkin (KMO) statistic and Bartlett’s test of sphericity. The number of factors to use was determined by parallel analysis in combination with inspection of the scree plot and the Kaiser criterion (eigenvalues > 1). Factor extraction was performed using principal axis factoring (PAF), which is recommended when the goal is to identify latent constructs rather than to maximize explained variance. Because we expected correlations between factors were present, an oblimin (oblique) rotation was used. Items with loadings ≥0.40 were considered salient. To assess the adequacy of the solution, we assessed the communalities, uniqueness values, and factor intercorrelations. Goodness of fit was evaluated using the chi-square test for model fit, the root mean square residual (RMSR), the Tucker–Lewis Index (TLI), and the root mean square error of approximation (RMSEA) with 90% confidence intervals. Multiple linear regression analyses were conducted to assess the independent relation between the DAS total score and its subscales with International IIEF general satisfaction scores. All models were adjusted for potential confounding variables, including duration of the relationship, prostate width, and the presence of relevant comorbidities (hypertension, ischemic cardiopathy, diabetes mellitus, obesity, and spinal and pelvic injuries). The number of variables in the model was chosen so that there were no fewer than ten subjects per variable to prevent overfitting. The initial model included the duration of relationship instead of age. Because a high correlation between the two would cause multicollinearity, if both variables would have stayed in the model, the coefficients could be destabilized. Since the distribution of age was large and potentially impactful on the DAS scores and IIEF, we chose variable age in the model, instead of the duration of relationship. Before performing regression analyses, key assumptions were assessed as follows: normality of residuals was evaluated visually using quantile–quantile plots, with Box–Cox transformations applied when needed; heteroskedasticity was assessed via scale–location plots and, where present, robust standard errors were used; multicollinearity among predictors was examined using variance inflation factors; and linearity between continuous predictors and outcomes was checked with component + residual plots. All statistical analyses were performed using the R environment for statistical computing and graphics (R Foundation for Statistical Computing, Vienna, Austria) version 4.3.2, and a two-tailed *p*-value < 0.05 was considered statistically significant [24].

During the preparation of this manuscript/study, the author(s) used ChatGPT (OpenAI, v4.0, April 2024) as a brainstorming tool to generate suggestions for perspectives to include in the Discussion Section. The authors have reviewed and edited the output and take full responsibility for the content of this publication.

## 3. Results

The study sample consisted of 94 participants, with a median age of 59 years, ranging from 21 to 88 years (Table 1). Most of the participants were married. The most common comorbidity was hypertension, followed at a long distance by diabetes mellitus. The largest part of the participants were not smokers. The clinical interpretation of these results suggests a moderate level of overall relationship functioning and sexual health challenges in this group. The DAS subscale scores indicate suboptimal relationship quality, with median scores across Affectional Expression (45), Consensus (55), Cohesion (54), Satisfaction (54), and Adaptation (55), suggesting preserved emotional connection and cooperation. Erectile dysfunction has a median duration of 12 months, and IIEF-15 has low median scores on the following domains: Erectile Function (13.5), Orgasmic Function (6), and Overall Satisfaction (4). These findings indicate clinically significant sexual dysfunction, although sexual desire (6) is relatively better preserved. The IPSSs (QoL question: 5; total: 9) show that the male population of our study had mild-to-moderate LUTS, which could affect sexual intercourse. These results suggest sexual distress and strain, which may impact intimacy and quality of life, even though the median relationship duration was 30 years.

**Figure 1:**
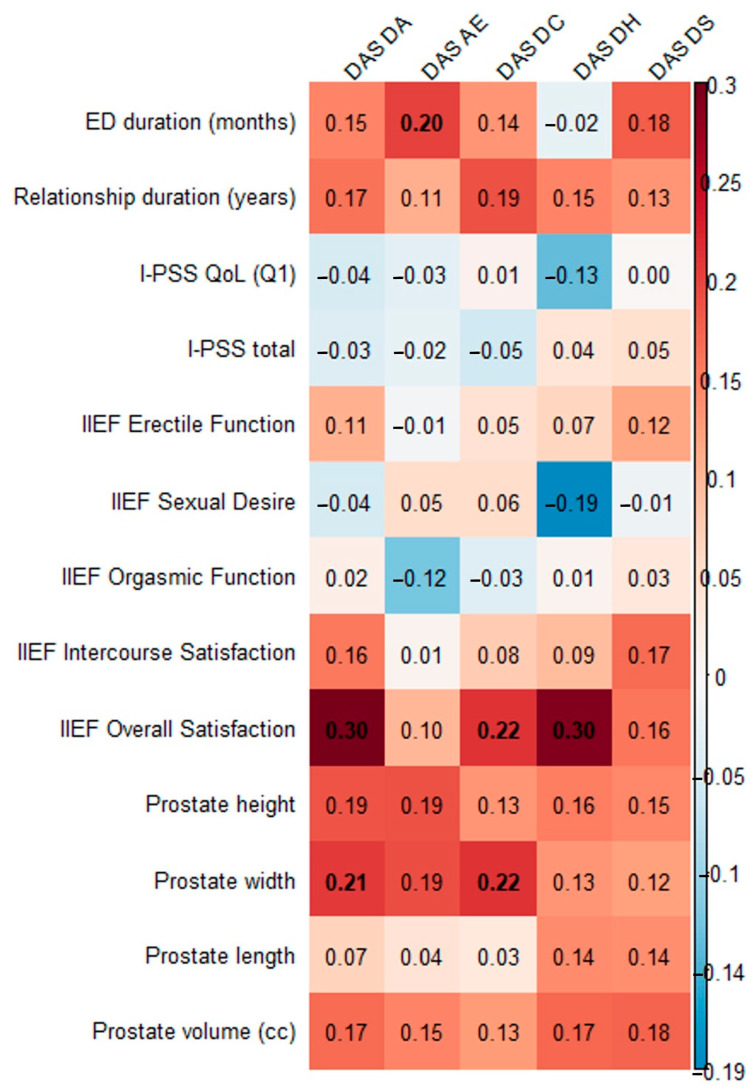
Correlations between DAS and I-PSS, IIEF, prostate dimensions, and duration of ED. ED, erectile dysfunction; I-PSS, International Prostate Symptom Score; QoL, quality of life; IIEF, International Index of Erectile Function; DAS, Dyadic Adjustment Scale; DC, Dyadic Consensus; DS, Dyadic Satisfaction; DH, Dyadic Cohesion; AE, Affectional Expression; DA, Dyadic Adaptation: bold numbers indicate statistically significant correlation coefficients.

We assessed the correlation of the DAS and its subscales with the duration of erectile dysfunction, the duration of the relationship, I-PSS, IIEF subscales, and prostate dimensions (Figure 1, Supplementary Table S1). Significant direct small proportional relationships were found between DAS AE and the length of erectile dysfunction, between IIEF general satisfaction and DAS DA, DAS DC, and DAS DH, and between prostate width and DAS DA and DAS DC.

Additionally, we checked the correlation between LUTS indicators (I-PSS QoL, I-PSS total, prostate height, width, length, volume) and ED indicators (ED duration, IIEF erectile function, sexual desire, orgasmic function, intercourse satisfaction, overall satisfaction), and almost all were significant (Supplementary Table S2), except IIEF Overall Satisfaction, which correlated only with I-PSS-total.

**Figure 2:**
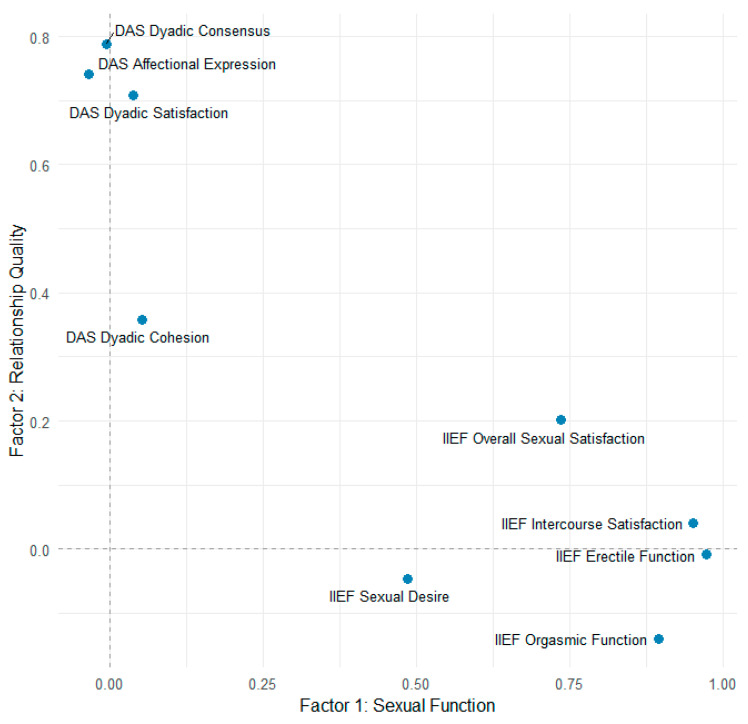
Factor loading plot from exploratory factor analysis of International Index of Erectile Function (IIEF) and Dyadic Adjustment Scale (DAS) subscales.

To investigate further the relations between DAS and IIEF an exploratory factor analysis was performed. The Kaiser–Meyer–Olkin measure was 0.79, indicating meritorious sampling adequacy. All subscales had values above 0.68 for MSA, indicating an acceptable range, except Dyadic Cohesion (MSA = 0.59). Bartlett’s test was significant (*p* < 0.001). These results suggested that the assumptions factor analysis are met. A parallel analysis found that two factors is an adequate solution. Exploratory factor analysis (principal axis factoring, oblimin rotation) found that two factors accounted for 59% of the variance (Factor 1 = 38%; Factor 2 = 21%). Factor 1 was composed of IIEF subscales: erectile function (loading 0.97), orgasmic function (0.90), intercourse satisfaction (0.95), sexual desire (0.49), and overall sexual satisfaction (0.74), suggesting a “sexual function” dimension. Factor 2 was composed of the DAS subscales of dyadic consensus (0.79), dyadic satisfaction (0.71), and affectional expression (0.74), suggesting a “relationship satisfaction” dimension. The dyadic cohesion had a weak loading (0.36) and a high uniqueness (0.87). This suggests it may represent a construct that differs more from the other subscales. The correlation between the two factors was close to 0 (r = 0.06), suggesting they are independent. The model fit results indicated that the model with two factors is adequate (TLI = 0.94; RMSEA = 0.09, 90% CI 0.04–0.14; RMSR = 0.05). The factor loading plot is presented in Figure 2. Factor 1 (“Sexual Function,” *x*-axis) was defined by erectile function, orgasmic function, intercourse satisfaction, sexual desire, and overall sexual satisfaction. Factor 2 (“Relationship Quality,” *y*-axis) was defined by dyadic consensus, affectional expression, and dyadic satisfaction. Dyadic cohesion showed weak association with the relational factor. Overall sexual satisfaction demonstrated modest cross-loading, suggesting it has both sexual and relational components. Since the axes are near-orthogonal, it implies the two factors are independent.

**Figure 3:**
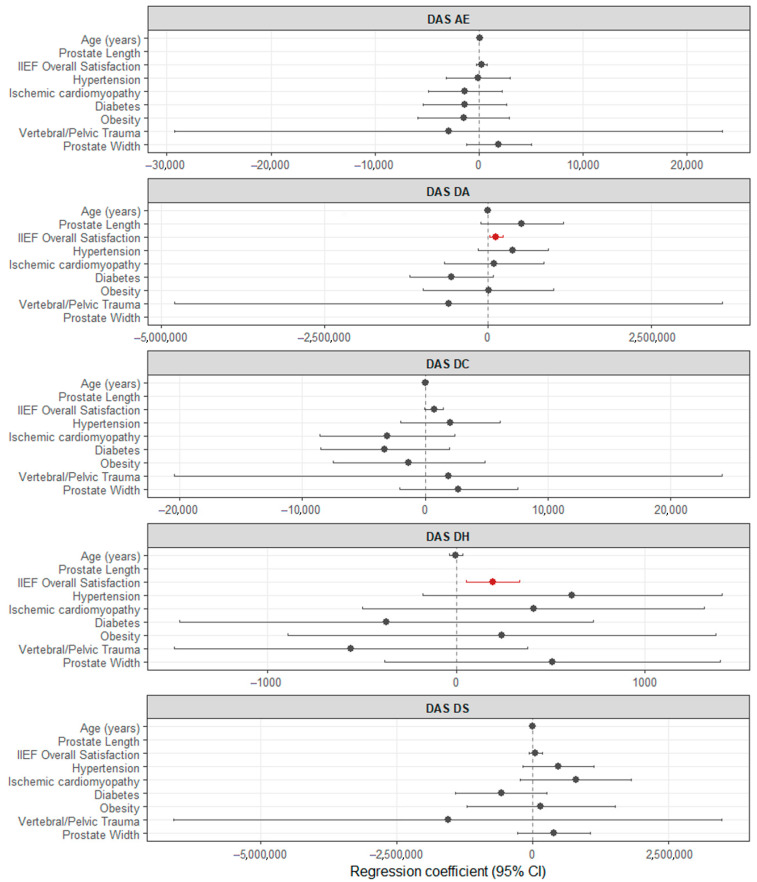
Forest plot of coefficients and 95% confidence intervals (CIs) of multiple linear regression models after Box–Cox transformation, predicting the dyadic adjustment scale (DAS) and subscales, with the international index of erectile function (IIEF) general satisfaction subscale adjusted for age, prostate width, and comorbidities (hypertension, ischaemic cardiopathy, diabetes mellitus, obesity, and spinal and pelvic injuries).

To investigate the robustness of the relationship between the DAS subscales with the IIEF general satisfaction subscale, we fit a multiple linear regression model for each DAS subscale as dependent variables regarding IIEF general satisfaction subscale and adjusted for age, prostate width, and comorbidities (Figure 3, Supplementary Table S3, while simple linear regression models are in Supplementary Table S4). Two of the initial univariate relationships remained significant after adjustment, indicating a direct proportional relationship between the IIEF general satisfaction subscale and DAS DA and DAS DH, while for DAS DC, the relationship was close to the significance level without reaching it. These results support the interpretation that the subjective appraisal of sexual life (Overall Satisfaction) functions as a “bridge” variable between sexual function and relational adjustment, consistent with the modest cross-loading of Overall Satisfaction observed in the factor analysis.

## 4. Discussion

This study assessed correlations between the DAS and its subscales and erectile dysfunction, prostate issues, and other clinical variables. We found a significant but small direct proportional correlation between DAS AE and erectile dysfunction duration, between IIEF general satisfaction and DAS DA, DC, and DH, and between prostate width and DAS DA and DC. Further multiple regression analyses adjusting for age, prostate width, and comorbidities showed that the associations between IIEF general satisfaction and DAS DA and DH remained significant, while the relationship with DAS DC was borderline.

Although the exploratory factor analysis demonstrated that sexual and relational domains generally load on distinct latent factors, the IIEF Overall Satisfaction subscale showed modest cross-loading on the relational factor. This pattern is consistent with our regression analyses, which indicated that Overall Satisfaction was positively associated with dyadic adaptation and cohesion, and marginally with consensus, even after adjustment for relationship duration, age, prostate width, and comorbidities. Taken together, these results suggest that while sexual functioning and relationship quality are largely independent constructs, the subjective appraisal of sexual life (captured by Overall Satisfaction) has a special overlap with relational well-being.

Affectional Expression was associated with ED duration but not with the IIEF domains. This suggests that physical expressions of affection may be more sensitive to the duration of erectile problems rather than to their current severity. Dyadic Consensus and Dyadic Cohesion correlated only with IIEF Overall Satisfaction, suggesting that perceptions of agreement and emotional bonding within the couple may be influenced by the subjective evaluation of sexual life rather than by the objective erectile capacity. Dyadic Satisfaction that indicates conflict resolution and general emotional stability, found no associations with sexual function measures. This underlines a relative independence of relational satisfaction from sexual performance. These results indicate that different aspects of relationship are differentially linked to sexual outcomes. Clinically, this underscores the need to assess both sexual and relational domains with care, as they may not be uniformly correlated and may require distinct therapeutic approaches.

These results resemble the existing literature regarding the association between sexual function and relationship quality. Przydacz et al. found that while LUTS were associated with ED and premature ejaculation, they did not significantly predict men’s overall sexual activity, suggesting that interpersonal factors, such as relationship quality, may impact this outcome [10]. Similarly, Maestre-Lorén et al. noted that erectile function and emotional status are critical factors of male sexual satisfaction in couples [25]. Several studies showed that psychological disorders such as depression and anxiety frequently coexist with LUTS and ED, intensifying their impact on male sexual health and satisfaction, thus supporting the significance of emotional intimacy and interpersonal connection [14,25,26]. Our findings are in agreement with these results and underscore the importance of evaluating dyadic adjustment alongside urological assessment in clinical practice [13,27,28].

Another study revealed that erectile function showed an independent association with both sexual satisfaction and quality of life across all age groups and served as a predictor for sexual satisfaction and overall health-related quality of life (HRQoL). The consistently low levels of sexual satisfaction observed across cohorts emphasize the essential role of assessing and addressing sexual health irrespective of patient age [29]. Sexual dysfunction, lower urinary tract symptoms (LUTS), and low relational satisfaction constitute a significant clinical concern for men with comorbidities such as type 2 diabetes mellitus, hypertension, benign prostatic hyperplasia (BPH), and ankylosing spondylitis (AS). These domains are closely interrelated: sexual dysfunction correlates significantly with depressive symptoms, poor glycemic control, and marital distress in diabetic patients and is further influenced by anxiety, disease chronicity, age, and functional impairment in individuals with AS [26,30,31,32].

The prevalence of LUTS is particularly high and associated with increased cardiovascular risk, as indicated by the Framingham–Wilson score in hypertensive males. Additional contributing factors include abdominal obesity, tobacco use, pathological electrocardiographic findings, erectile dysfunction, and the use of hypouricemic agents. Not smoking and the use of diuretics present a lower risk of LUTS. In BPH-related LUTS, the severity of symptoms correlates with elevated frequency of metabolic syndrome (MetS) and more severe erectile dysfunction. Prostate volume is inversely associated with International Index of Erectile Function (IIEF-5) scores [31,33,34]. These findings underscore the importance of early identification and comprehensive management of sexual health disturbances. A multidisciplinary approach—integrating urological, metabolic, cardiovascular, and psychological assessment—may enhance clinical outcomes and improve the quality of life in affected male patients. Our study observed a correlation between prostate width and some of the DAS subscales, but further validation of these anatomical findings is required. One large study, with a total of 943 patients, showed a significant correlation between prostate length and the severity of LUTS and ED [35].

Erectile dysfunction (ED), defined as the inability to obtain or maintain an erection for satisfactory sexual intercourse, often extends its impact on the partner, significantly affecting intimacy. Female partners of men with ED frequently report sexual distress and relationship conflict, which may lead to the onset or persistence of the dysfunction itself, treatment compliance, and even failure. Therefore, a comprehensive assessment that includes the partner is essential to accurately identify contributing relationship dynamics and optimize prognosis [36]. Unfortunately, clinical environments in many settings are not consistently structured to facilitate partner involvement, thus limiting understanding of the dyadic functioning. A more holistic approach to ED management should recognize the interpersonal dimensions of sexual health and promote partner participation throughout the diagnostic and treatment process [37,38]. Addressing relationship context through couples/sex therapy may improve the sexual satisfaction of both partners, adherence to lifestyle changes, medical therapies, and overall quality of life. Therefore, collaborative, multidisciplinary (between the urologist and the psychotherapist and occasionally the endocrinologist) therapeutic decision making and couple-based approaches may yield comprehensive and sustainable improvements in the management of ED and LUTS. Future randomized controlled trials with couple-based interventions should be designed to verify whether these observations would help more than male-only interventions.

Since the age range of the participants was wide and could have be correlated with LUTS, IIEF and DAS, we included age in the multilinear model to control for its effect and limit the confounding bias induced by age.

### Limitations

This study has several limitations. The cross-sectional design allows for sustaining association but cannot sustain causality. Although data were collected prospectively, temporal relationships cannot be inferred. The bicenter nature of the sample indicates that it may not be fully representative of all men with prostate-related conditions, limiting the generalizability of the findings. DAS, IIEF-15, and IPSS are self-reported measures that can be subject to response and recall bias and social desirability bias, potentially affecting the accuracy of responses regarding sensitive issues such as sexual satisfaction and relationship dynamics. Other confounding factors not included in the model could be the following: the participant’s mental health status, characteristics of their partner (such as age, health, and relationship satisfaction), and the side effects of medications that may influence sexual performance or psychological well-being. Given the relatively small sample size (*n* = 94), the findings of this study may not be generalizable to the Romanian population. The effect sizes are small, and this limits the clinical relevance. The lack of partner-reported outcomes further limits the understanding of dyadic functioning.

The sample was predominantly heterosexual (98.9%) with one same-sex couple, precluding subgroup analyses by sexual orientation. The findings may not generalize to sexual-minority men or gender-diverse populations. Future studies will include SAGER (Sex and Gender Equity in Research) Guidelines-aligned measures (sex assigned at birth, gender identity, partner gender, sexual orientation, relationship structure) and be powered for subgroup analyses.

Being an observational study, confounding factors, such as psychological status or partner characteristics, may have influenced the results.

## 5. Conclusions

Our findings highlight that general sexual satisfaction, as measured by the IIEF-15, had a small but independent association with higher affective expressions (AEs), dyadic cohesion (DH), and dyadic consensus (DC) in couples as key domains of dyadic adjustment, regardless of relationship duration, prostate width, and comorbidities. Prostate width had a small negative correlation with dyadic adjustment (DA) and dyadic consensus (DC). These results emphasize the importance of considering sexual satisfaction in the context of relationship quality and, therefore, involving the female partner in the assessment and treatment of ED, and also suggest that psychological approaches used for improving sexual health may have a positive impact on couple adjustment, particularly in men with LUTS. The need for complex management of sexually related issues is a desideratum that involves the urologist, endocrinologist, and also the psychotherapist.

## Figures and Tables

**Table 1:** Participants’ characteristics.

Characteristic	
Age (years), median (IQR)	59 (50–67.75)
Marital Status	
Married:	70/94 (74.46)
Divorced:	4/94 (4.26)
Widowed:	4/94 (4.26)
Unmarried:	16/94 (17.02)
Comorbidities	
Hypertension	58/94 (61.7)
Ischemic cardiomyopathy	10/94 (10.64)
Diabetes	16/94 (17.02)
Obesity	10/94 (10.64)
Vertebral/pelvic trauma	2/94 (2.13)
Cigarettes Frequency	
0	71/94 (75.53)
<5/day:	4/94 (4.26)
5–10/day:	9/94 (9.57)
21–30/day:	10/94 (10.64)
DAS Dyadic Adaption, median (IQR)	55 (49.25–60)
DAS Affectional Expression, median (IQR)	45 (40–50)
DAS Dyadic Consensus, median (IQR)	55 (48–63)
DAS Dyadic Cohesion, median (IQR)	54 (44–62)
DAS Dyadic Satisfaction, median (IQR)	54 (48.5–58)
ED duration (months), median (IQR)	12 (8–20.75)
Relationship duration (years), median (IQR)	30 (11–42)
I-PSS QoL question 1, median (IQR)	5 (2–22.5)
I-PSS total, median (IQR)	9 (3.25–17.75)
IIEF Erectile Function, median (IQR)	13.5 (3–20)
IIEF Sexual Desire, median (IQR)	6 (5–8)
IIEF Orgasmic function, median (IQR)	6 (0–8.75)
IIEF Intercourse Satisfaction, median (IQR)	6 (0–10)
IIEF Overall Satisfaction, median (IQR)	4 (2.25–6)
Prostate height, median (IQR)	4.65 (4.23–5)
Prostate width, median (IQR)	4.6 (4.3–4.9)
Prostate length, median (IQR)	4.85 (4.5–5.2)
Prostate volume (cc), median (IQR)	53.82 (43.52–62.79)

IQR, interquartile range; ED, erectile dysfunction; I-PSS, International Prostate Symptom Score; QoL, quality of life; IIEF, International Index of Erectile Function; DAS, Dyadic Adjustment Scale.

## Data Availability

The dataset is available upon request from the authors.

## Supplementary Materials

The following supporting information can be downloaded at: https://www.mdpi.com/article/10.3390/medicina61091590/s1, Figure S1. Scree plot with Parallel analysis (PA) overlay. Table S1. Correlations between DAS and I-PSS, IIEF, prostate dimensions, and duration of ED. Table S2. Spearman correlations between I-PSS QoL, I-PSS total, prostate height, width, length, volume) and ED indicators. Table S3. Multiple linear regression models predicting the dyadic adjustment scale (DAS) and subscales, with the international index of erectile function (IIEF), general satisfaction subscale, adjusted for age, prostate width, and comorbidities (hypertension, ischaemic cardiopathy, diabetes mellitus, obesity, and spinal and pelvic injuries). Table S4. Simple linear regression models predicting the dyadic adjustment scale (DAS) and subscales, with the international index of erectile function (IIEF), general satisfaction subscale, as well as for age, prostate width, and comorbidities (hypertension, ischaemic cardiopathy, diabetes mellitus, obesity, and spinal and pelvic injuries).

## Abbreviations

The following abbreviations are used in this manuscript:

CI	Confidence Interval
IQR	Interquartile Range
ED	Erectile Dysfunction
I-PSS	International Prostate Symptom Score
QoL	Quality of Life
IIEF	International Index of Erectile Function
DAS	Dyadic Adjustment Scale
DC	Dyadic Consensus
DS	Dyadic Satisfaction
DH	Dyadic Cohesion
AE	Affectional Expression
DA	Dyadic Adaptation
