# Supplementary material for: The Relationship Between Erectile Dysfunction and Dyadic Adjustment, Couple Relationship Quality, and Intimacy: A Cross-Sectional Study
Source: Medicina (Kaunas). 2025 Sep 3;61(9):1590. doi: 10.3390/medicina61091590 (PMC12471714; doi:10.3390/medicina61091590)
Supplement: Supplementary file 1 [file medicina-61-01590-s001.zip › medicina-3835149-supplementary.pdf]

Supplementary Table S1. Correlations between DAS and I-PSS, IIEF, prostate dimensions, and duration of ED.

Characteristics	DAS DA	DAS AE	DAS DC	DAS DH	DAS DS
ED duration (months)	0.15 (0.152)	0.2 (0.049)	0.14 (0.192)	-0.02 (0.857)	0.18 (0.076)
Relationship duration (years)	0.17 (0.111)	0.11 (0.29)	0.19 (0.063)	0.15 (0.15)	0.13 (0.197)
I-PSS QoL question 1	-0.04 (0.673)	-0.03 (0.799)	0.01 (0.904)	-0.13 (0.212)	0 (0.99)
I-PSS total	-0.03 (0.748)	-0.02 (0.847)	-0.05 (0.634)	0.04 (0.7)	0.05 (0.629)
IIEF Erectile Function	0.11 (0.311)	-0.01 (0.94)	0.05 (0.655)	0.07 (0.531)	0.12 (0.247)
IIEF Sexual Desire	-0.04 (0.674)	0.05 (0.601)	0.06 (0.586)	-0.19 (0.06)	-0.01 (0.89)
IIEF Orgasmic function	0.02 (0.847)	-0.12 (0.249)	-0.03 (0.758)	0.01 (0.92)	0.03 (0.743)
IIEF Intercourse Satisfaction	0.16 (0.126)	0.01 (0.911)	0.08 (0.462)	0.09 (0.363)	0.17 (0.1)
IIEF Overall Satisfaction	0.3 (0.003)	0.1 (0.329)	0.22 (0.035)	0.3 (0.004)	0.16 (0.121)
Prostate height	0.19 (0.07)	0.19 (0.064)	0.13 (0.2)	0.16 (0.131)	0.15 (0.156)
Prostate width	0.21 (0.041)	0.19 (0.061)	0.22 (0.037)	0.13 (0.197)	0.12 (0.231)
Prostate length	0.07 (0.51)	0.04 (0.685)	0.03 (0.762)	0.14 (0.164)	0.14 (0.178)
Prostate volume (cc)	0.17 (0.101)	0.15 (0.153)	0.13 (0.219)	0.17 (0.096)	0.18 (0.091)

The data represents Spearman correlation coefficients (ρ) with p-value in the brackets; ED, erectile dysfunction; I-PSS, International Prostate Symptom Score; QoL, quality of life; IIEF, International Index of Erectile Function; DAS, Dyadic Adjustment Scale; DC, Dyadic Consensus; DS, Dyadic Satisfaction; DH, Dyadic Cohesion; AE, Affectional Expression; DA, Dyadic Adaptation.

Supplementary Table S2. Spearman correlations between I-PSS QoL, I-PSS total, prostate height, width, length, volume) and ED indicators.

	I-PSS QoL	I-PSS total	Prostate height	Prostate width	Prostate length	Prostate volume (cc)
**ED duration (months)**	0.14 (0.187 / 1.0000)	0.39 (< 0.001 / 0.0050)	0.36 (< 0.001 / 0.0143)	0.3 (0.004 / 0.1091)	0.27 (0.009 / 0.2338)	0.36 (< 0.001 / 0.0126)
**IIEF Erectile Function**	0.21 (0.038 / 0.4540)	-0.31 (0.002 / 0.0742)	-0.33 (0.001 / 0.0425)	-0.23 (0.024 / 0.4364)	-0.4 (< 0.001 / 0.0034)	-0.35 (< 0.001 / 0.0245)
**IIEF Sexual Desire**	0.21 (0.041 / 0.4540)	-0.24 (0.021 / 0.4364)	-0.29 (0.005 / 0.1320)	-0.23 (0.027 / 0.4364)	-0.29 (0.005 / 0.1476)	-0.29 (0.004 / 0.1320)
**IIEF Orgasmic function**	0.27 (0.01 / 0.2338)	-0.33 (0.001 / 0.0374)	-0.41 (< 0.001 / 0.0016)	-0.29 (0.005 / 0.1476)	-0.47 (< 0.001 / <.0001)	-0.45 (< 0.001 / 0.0003)

**IIEF Intercourse Satisfaction**	0.21 (0.041 / 0.4540)	-0.24 (0.019 / 0.4124)	-0.35 (< 0.001 / 0.0185)	-0.24 (0.021 / 0.4364)	-0.4 (< 0.001 / 0.0036)	-0.37 (< 0.001 / 0.0116)
**IIEF Overall Satisfaction**	0.07 (0.528 / 1.0000)	-0.23 (0.023 / 0.4364)	-0.08 (0.426 / 1.0000)	-0.09 (0.397 / 1.0000)	-0.19 (0.069 / 0.6221)	-0.14 (0.18 / 1.0000)

Data is presented as Spearman correlation coefficient (P-value unadjusted / P-value adjusted for multiple correlations using Holm method); ED, erectile dysfunction; I-PSS, International Prostate Symptom Score; QoL, quality of life; IIEF, International Index of Erectile Function.

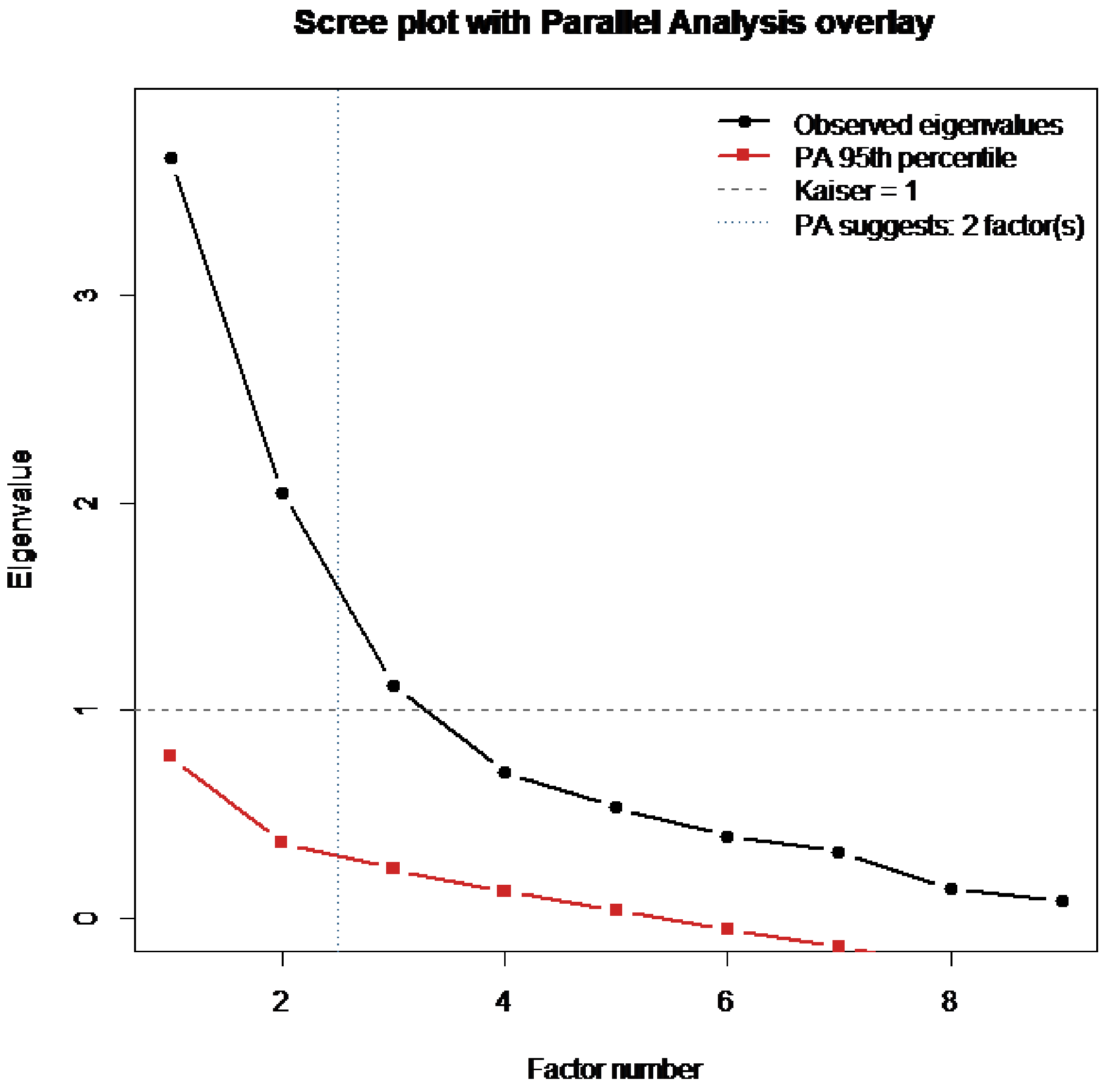

Supplementary Figure S1. Scree plot with Parallel analysis (PA) overlay.

Supplementary Table S3. Multiple linear regression models predicting the dyadic adjustment scale (DAS) and subscales, with the international index of erectile function (IIEF), general satisfaction subscale, adjusted for age, prostate

width, and comorbidities (hypertension, ischaemic cardiopathy, diabetes mellitus, obesity, and spinal and pelvic injuries).

Dependent variable	Independent variable	Coefficient	95% CI lower bound	95% CI upper bound	P value	VIF
DAS DA						
	Age (years)	2793.1	-21280.2	26866	0.8181	2
	Prostate Width	519798.3	-110504.6	1150101	0.1048	1.5
	IIEF Overall Satisfaction	126201.3	27912.4	224490	0.0125	1.2
	Hypertension	384670.9	-153712.4	923054	0.1591	1.3
	Ischemic cardiomyopathy	90253.9	-671551.5	852059	0.8143	1.2
	Diabetes	-555758.2	-1197789.3	86273	0.0889	1.3
	Obesity	5196.7	-987313.7	997707	0.9917	1.2
	Vertebral/Pelvic Trauma	-605198.7	-4792401.9	3582004	0.7745	1.1
DAS AE						
	Age (years)	63.8	-44.7	172	0.2456	2
	Prostate Width	1908.6	-1207.9	5025	0.2267	1.5
	IIEF Overall Satisfaction	293.6	-222.5	810	0.2612	1.2
	Hypertension	-77.2	-3125.0	2971	0.9600	1.3
	Ischemic cardiomyopathy	-1357.7	-4900.0	2185	0.4481	1.2
	Diabetes	-1357.4	-5379.8	2665	0.5041	1.3
	Obesity	-1487.2	-5909.9	2936	0.5056	1.2
	Vertebral/Pelvic Trauma	-2933.2	-29225.9	23359	0.8250	1.1
DAS DC						
	Age (years)	69.2	-111.8	250	0.4492	2
	Prostate Width	2741.5	-2081.6	7565	0.2616	1.5
	IIEF Overall Satisfaction	754.2	-3.9	1512	0.0512	1.2
	Hypertension	2077.6	-1984.5	6140	0.3121	1.3
	Ischemic cardiomyopathy	-3085.1	-8591.5	2421	0.2684	1.2
	Diabetes	-3265.1	-8503.1	1973	0.2186	1.3
	Obesity	-1306.7	-7496.3	4883	0.6757	1.2
	Vertebral/Pelvic Trauma	1883.6	-20387.4	24155	0.8669	1.1
DAS DH						
	Age (years)	-2.3	-37.8	33	0.8994	2
	Prostate Width	511.2	-379.7	1402	0.2571	1.5

	IIEF Overall Satisfaction	194.2	52.1	336	0.0080	1.2
	Hypertension	615.6	-176.4	1408	0.1259	1.3
	Ischemic cardiomyopathy	408.7	-498.3	1316	0.3728	1.2
	Diabetes	-371.4	-1469.9	727	0.5033	1.3
	Obesity	240.3	-895.5	1376	0.6751	1.2
	Vertebral/Pelvic Trauma	-557.8	-1494.2	379	0.2396	1.1
DAS DS						
	Age (years)	-5294.8	-32870.7	22281	0.7036	2
	Prostate Width	387742.9	-276629.3	1052115	0.2491	1.5
	IIEF Overall Satisfaction	49985.4	-69886.8	169858	0.4094	1.2
	Hypertension	468646.8	-190983.2	1128277	0.1614	1.3
	Ischemic cardiomyopathy	789054.5	-236745.0	1814854	0.1299	1.2
	Diabetes	-581926.0	-1417266.1	253414	0.1696	1.3
	Obesity	148104.4	-1218099.2	1514308	0.8299	1.2
	Vertebral/Pelvic Trauma	-1561315.7	-6589586.2	3466955	0.5386	1.1

CI, confidence interval; IIEF, International Index of Erectile Function; DAS, Dyadic Adjustment Scale; DC, Dyadic Consensus; DS, Dyadic Satisfaction; DH, Dyadic Cohesion; AE, Affectional Expression; DA, Dyadic Adaptation; VIF, variance inflation factors.

Supplementary Table S4. Simple linear regression models predicting the dyadic adjustment scale (DAS) and subscales, with the international index of erectile function (IIEF), general satisfaction subscale, as well as for age, prostate width, and comorbidities (hypertension, ischaemic cardiopathy, diabetes mellitus, obesity, and spinal and pelvic injuries).

Dependent variable	Independent variable	Coefficient	95% CI lower bound	95% CI upper bound	P value
DAS DA					
	Age (years)	0.12	-0.026	0.27	0.106
	Prostate Length	4.78	0.841	8.71	0.018
	IIEF Overall Satisfaction	1.01	0.228	1.79	0.012
	Hypertension	3.29	-0.847	7.42	0.118
	Ischemic cardiomyopathy	3.02	-3.554	9.60	0.364
	Diabetes	-3.69	-9.055	1.67	0.175
	Obesity	-1.90	-8.495	4.69	0.568
	Vertebral/Pelvic Trauma	-5.11	-19.181	8.96	0.473
DAS AE					

	Age (years)	0.11	-0.055	0.27	0.190
	Prostate Width	3.88	-0.498	8.26	0.082
	IIEF Overall Satisfaction	0.57	-0.313	1.45	0.204
	Hypertension	0.57	-4.023	5.17	0.805
	Ischemic cardiomyopathy	0.90	-6.348	8.14	0.806
	Diabetes	-3.15	-9.057	2.76	0.293
	Obesity	-3.13	-10.350	4.09	0.391
	Vertebral/Pelvic Trauma	-7.46	-22.866	7.95	0.339
DAS DC					
	Age (years)	0.13	-0.0329	0.29	0.117
	Prostate Width	4.57	0.2458	8.89	0.039
	IIEF Overall Satisfaction	0.86	-0.0043	1.73	0.051
	Hypertension	3.10	-1.4255	7.62	0.177
	Ischemic cardiomyopathy	-0.99	-8.1864	6.21	0.786
	Diabetes	-3.93	-9.7780	1.93	0.186
	Obesity	-2.44	-9.6263	4.74	0.501
	Vertebral/Pelvic Trauma	3.18	-12.1874	18.56	0.682
DAS DH					
	Age (years)	0.091	-0.094	0.28	0.331
	Prostate Width	4.550	-0.416	9.52	0.072
	IIEF Overall Satisfaction	1.401	0.434	2.37	0.005
	Hypertension	3.804	-1.355	8.96	0.146
	Ischemic cardiomyopathy	5.126	-3.033	13.28	0.215
	Diabetes	-2.720	-9.446	4.01	0.424
	Obesity	0.202	-8.025	8.43	0.961
	Vertebral/Pelvic Trauma	-3.391	-20.956	14.17	0.702
DAS DS					
	Age (years)	0.084	-0.054	0.22	0.228
	Prostate Width	2.536	-1.188	6.26	0.179
	IIEF Overall Satisfaction	0.471	-0.274	1.22	0.212
	Hypertension	2.875	-0.962	6.71	0.140
	Ischemic cardiomyopathy	5.338	-0.684	11.36	0.082
	Diabetes	-1.678	-6.689	3.33	0.508
	Obesity	-1.040	-7.159	5.08	0.736
	Vertebral/Pelvic Trauma	-11.065	-23.945	1.81	0.091

CI, confidence interval; IIEF, International Index of Erectile Function; DAS, Dyadic Adjustment Scale; DC, Dyadic Consensus; DS, Dyadic Satisfaction; DH, Dyadic Cohesion; AE, Affectional Expression; DA, Dyadic Adaptation.
